# A multicenter retrospective study of patients treated in the thalamus with responsive neurostimulation

**DOI:** 10.3389/fneur.2023.1202631

**Published:** 2023-09-08

**Authors:** Madeline C. Fields, Onome Eka, Cristina Schreckinger, Patricia Dugan, Wael F. Asaad, Andrew S. Blum, Katie Bullinger, Jon T. Willie, David E. Burdette, Christopher Anderson, Imran H. Quraishi, Jason Gerrard, Anuradha Singh, Kyusang Lee, Ji Yeoun Yoo, Saadi Ghatan, Fedor Panov, Lara V. Marcuse

**Affiliations:** ^1^Department of Neurology, Icahn School of Medicine at Mount Sinai, New York, NY, United States; ^2^St. Mary’s Health Care System, Athens, GA, United States; ^3^Department of Neurology, Langone Medical Center, New York University, New York, NY, United States; ^4^Department of Neurosurgery, Warren Alpert Medical School, Brown University, Providence, RI, United States; ^5^Department of Neurology, Warren Alpert Medical School, Brown University, Providence, RI, United States; ^6^Department of Neurology, School of Medicine, Emory University, Atlanta, GA, United States; ^7^Department of Neurosurgery, School of Medicine, Washington University in St Louis, St. Louis, MO, United States; ^8^Department of Neurosciences, Corewell Health, Grand Rapids, MI, United States; ^9^Department of Neurology, Medical College of Wisconsin, Milwaukee, WI, United States; ^10^Department of Neurology, School of Medicine, Yale University, New Haven, CT, United States; ^11^Department of Neurosurgery, School of Medicine, Yale University, New Haven, CT, United States; ^12^Department of Neurosurgery, Icahn School of Medicine at Mount Sinai, New York, NY, United States

**Keywords:** centromedian nucleus of thalamus, anterior thalamic nucleus, neuromodulation, responsive neurostimulation (RNS), drug-resistant epilepsy (DRE), epilepsy surgery

## Abstract

**Introduction:**

For drug resistant epilepsy patients who are either not candidates for resective surgery or have already failed resective surgery, neuromodulation is a promising option. Neuromodulatory approaches include responsive neurostimulation (RNS), deep brain stimulation (DBS), and vagal nerve stimulation (VNS). Thalamocortical circuits are involved in both generalized and focal onset seizures. This paper explores the use of RNS in the centromedian nucleus of the thalamus (CMN) and in the anterior thalamic nucleus (ANT) of patients with drug resistant epilepsy.

**Methods:**

This is a retrospective multicenter study from seven different epilepsy centers in the United States. Patients that had unilateral or bilateral thalamic RNS leads implanted in the CMN or ANT for at least 6 months were included. Primary objectives were to describe the implant location and determine changes in the frequency of disabling seizures at 6 months, 1 year, 2 years, and > 2 years. Secondary objectives included documenting seizure free periods, anti-seizure medication regimen changes, stimulation side effects, and serious adverse events. In addition, the global clinical impression scale was completed.

**Results:**

Twelve patients had at least one lead placed in the CMN, and 13 had at least one lead placed in the ANT. The median baseline seizure frequency was 15 per month. Overall, the median seizure reduction was 33% at 6 months, 55% at 1 year, 65% at 2 years, and 74% at >2 years. Seizure free intervals of at least 3 months occurred in nine patients. Most patients (60%, 15/25) did not have a change in anti-seizure medications post RNS placement. Two serious adverse events were recorded, one related to RNS implantation. Lastly, overall functioning seemed to improve with 88% showing improvement on the global clinical impression scale.

**Discussion:**

Meaningful seizure reduction was observed in patients who suffer from drug resistant epilepsy with unilateral or bilateral RNS in either the ANT or CMN of the thalamus. Most patients remained on their pre-operative anti-seizure medication regimen. The device was well tolerated with few side effects. There were rare serious adverse events. Most patients showed an improvement in global clinical impression scores.

## 1. Introduction

Neuromodulation is now recognized as an epilepsy surgery treatment alternative for drug resistant epilepsy (DRE) patients who are not resective or ablative surgical candidates. Candidates for neuromodulation include those with multifocal epilepsy, seizure foci in eloquent cortex, as well as those with generalized epilepsy. Vagal nerve stimulation (VNS), responsive neurostimulation (RNS), and deep brain stimulation (DBS) are neuromodulatory approaches used for DRE. VNS and DBS provide a continuous or pre-fixed electrical stimulation cycle. In VNS, extra stimulation can be delivered with the patient magnet or in response to tachycardia. On the other hand, RNS is a closed-loop device activated by abnormal electrocorticography patterns in or near the seizure focus (1). Because epilepsy is thought to involve corticothalamic networks, DBS and RNS have been increasingly applied to the thalamus (1–20).

Discussion of the thalamus in epilepsy dates to Wilder Penfield in the 1950s (21–23). Penfield posited that the thalamus was involved at the onset of absence and generalized tonic clonic seizures and may be rapidly engaged in seizures of temporal and frontal onset. Although resective or ablative epilepsy surgery is the best chance for cure, not every patient is a candidate as the seizure onset zone may be more extensive or in eloquent cortex. In the pivotal clinical trial, the RNS device treated patients with two seizure foci or with a seizure onset in eloquent cortex with electrodes as close to the onset zone as possible (24). Interrupting the seizure via the thalamic network responsively is a novel concept and while implemented at multiple level four epilepsy centers, has not been written about extensively.

Neuromodulation has been used in several nuclei of the thalamus to interrupt and modulate the neural networks with the objective of seizure reduction. The centromedian nucleus of the thalamus (CMN) is involved in wakefulness and has broad cortical projections. This network is related to seizure initiation, propagation and loss of consciousness (24). The anterior nucleus of the thalamus (ANT) is a key node in the limbic (circuit of Papez) and frontotemporal networks (25). DBS placement in the ANT or CMN is thought to modulate corticothalamic pathways (20, 26). A potential limitation of DBS is the continuous electrical stimulation regardless of the patient’s ictal state (27). Although less readily available to the practicing clinician, local field potential power spectral analysis is now available with DBS systems and can allow for assessment of seizures over time. RNS on the other hand provides stimulation that recognizes seizure patterns before delivering stimulation and keeps a fairly detailed record of seizure events (24).

We retrospectively reviewed and characterized patients treated with thalamic RNS across seven centers. Our main objectives were to describe clinical changes in disabling seizures, the thalamic nuclei implanted and whether the approach was bilateral or unilateral. Our secondary objectives were to assess seizure free periods of greater than 3 months, anti-seizure medication (ASM) regimen changes, stimulation side effects, serious adverse events, and the overall global clinical impression.

## 2. Methods

**Figure 1:**
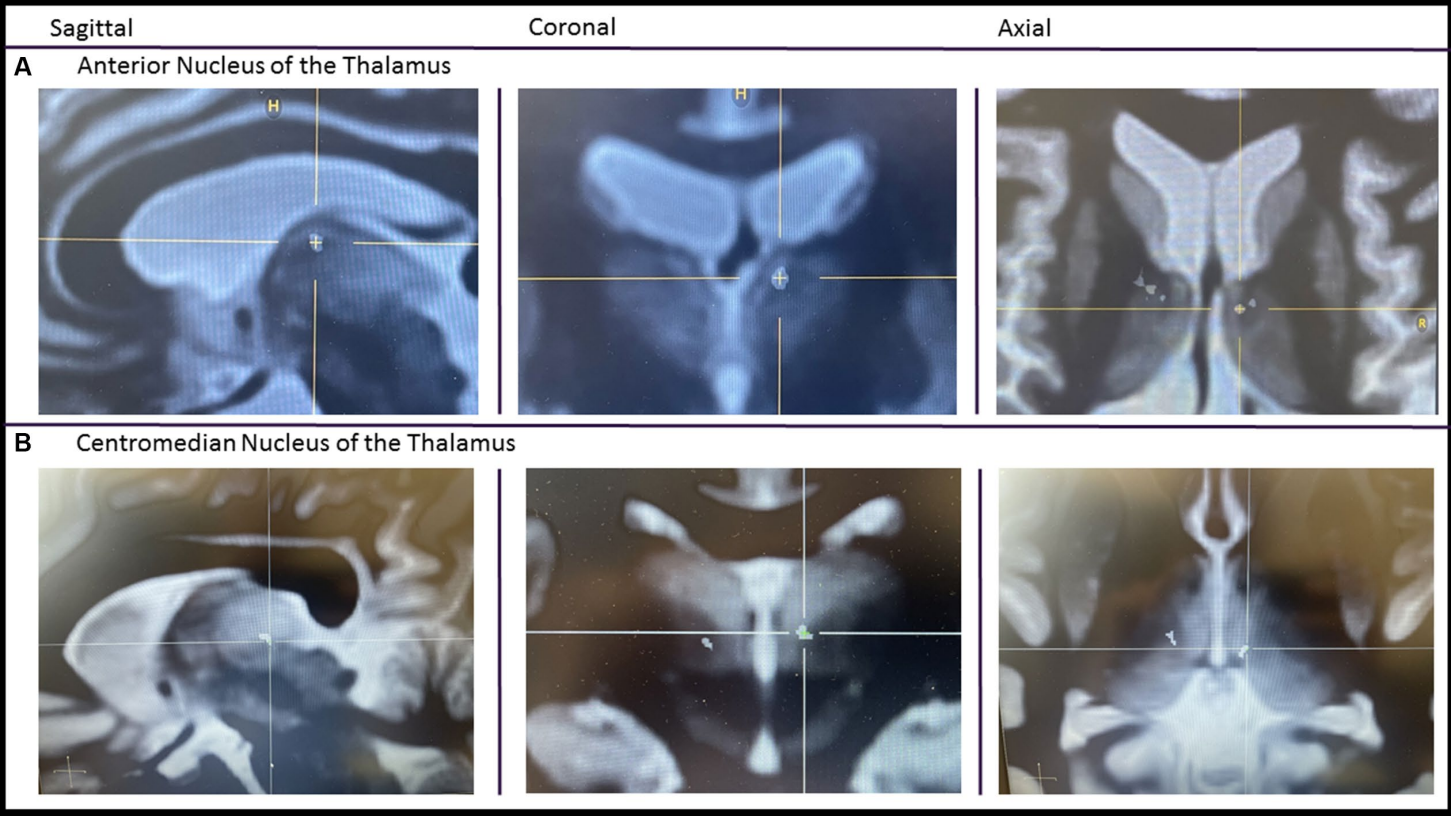
Imaging of thalamic RNS. A pre-op FGATIR MRI used for targeting is fused with a post-op volumetric CT. After fusion, the CT scan is made translucent except for the contacts. Imaging centered on the most internal contact of the left anterior thalamic lead **(A)** and centromedian lead **(B)**.

A retrospective multicenter study across seven epilepsy centers in the United States was performed including Mount Sinai Hospital, NYU Langone Medical Center, Emory University, Yale University, Brown University/Rhode Island Hospital, Corewell Health, and Medical College of Wisconsin. A waiver of informed consent and a HIPPA waiver were obtained from the IRB at all sites granting permission to access medical records for observational research purposes. Inclusion criteria included any patients that received at least one thalamic RNS lead at least 6 months prior to February of 2020. At all centers, the thalamic nuclei were targeted based on volumetric T1 MPRAGE and/or FGATIR MRI sequences. Placement was often confirmed with a post-op volumetric CT (Figure 1). Patients that received the RNS system for off-label indications, for example a pediatric patient or patients with generalized epilepsy, were not excluded from the study. Primary objectives were to describe the implant location and determine changes in the frequency of disabling seizures at 6 months, 1 year, 2 years, and at the >2 year visit. Secondary objectives included documenting seizure free periods of greater than 3 months, ASM regimen changes, stimulation side effects, and serious adverse events. In addition, the global cognitive impression scale (GCI-I) was performed.

Patient demographics were collected including gender, age at implant, duration with epilepsy, and etiology of the epilepsy. Comprehensive presurgical workup prior to the RNS implant was collected including MRI, scalp EEG, and intracranial EEG data when performed. Patient’s surgical history was explored as well as prior or concurrent use of other neuromodulatory treatments like VNS. Information regarding RNS implantation and therapy was collected for each patient. The specific nucleus of the thalamus and other targets of stimulation and detection were recorded. Descriptive statistics were used for analysis. Seizure reduction was estimated according to patient and clinician subjective report at each follow-up visit. Several cases included here were described in previous reports (1–3, 8).

## 3. Results

### 3.1. Patient demographics

A total of 25 DRE patients (14 M, 11F) were enrolled in the study. The age at implant ranged from 9 to 53 years with a mean age of 27.2 (Table 1). All 25 subjects had been treated with RNS for a minimum of 6 months, 20 patients for 1 year, 18 patients for 2 years, and 10 patients for >2 years. Of note, no patients were lost to follow up. Thirteen (52%) had previous epilepsy surgery (six temporal lobe resections, two corpus callosotomies, two frontal resections, one frontal/parietal resection, one frontal/temporal resection, and one hemispheric resection). Duration of epilepsy ranged from 7 to 39 years (mean duration 19.4 years). Ten patients had no known cause for their epilepsy, 11 had a structural cause, three had a genetic cause, and one had an infectious cause. Of the structural causes, four patients had polymicrogyria, two patients had unilateral mesial temporal sclerosis (MTS), one patient had bilateral MTS, one patient had periventricular nodular heterotopia, one patient had tuberous sclerosis, one patient had post traumatic injury, and one patient had Dyke-Davidoff-Masson syndrome (Table 1).

**Table 1:** Participant data.

Gender age at RNS implant	Epilepsy duration	Previous respective epilepsy surgery Y/N	Previous VNS Y/N	Epilepsy type (Focal/Generalized)	Etiology (description)	RNS location
M40	7	N	Y	Focal	Infectious (Coxsackie B meningoencephalitis)	B CMN
F26	15	N	Y	Generalized	Genetic (JME with Jeavon’s syndrome)	B ANT
F30	22	N	Y	Generalized	Genetic (JME)	R ANT, L F
M53	21	Y (R ATL)	Y	Focal	Structural (Polymicrogyria)	L ANT, L HCP
F36	36	Y (L ATL)	Y	Focal	Structural (Bilateral MTS)	R ANT, R HCP
M29	10	N	N	Focal	Structural (L MTS)	L ANT, L HCP
F38	37	Y (R selective medial temporal resection)	Y	Focal	Structural (L MTS)	L ANT, L HCP
M32	20	N	N	Focal	Structural (Periventricular nodular heterotopia)	R CMN, R P
M9	9	Y (CC)	N	Focal	Unknown	L CMN, R F
F24	24	N	Y	Generalized	Unknown	R ANT, R SMA
F14	14	Y (R hemispheric resection)	Y	Generalized	Unknown (LGS)	L CMN, R F
M16	16	Y (see *)	Y	Focal	Unknown	L ANT, R T
M31	27	N	Y	Focal	Structural (Dyke-Davidoff-Masson Syndrome)	B CMN
M12	4	N	N	Generalized	Genetic (Dup 15q, LGS)	B CMN
M19	11	N	N	Focal	Structural (Polymicrogyria)	R ANT, R T
F10	10	Y (CC)	N	Focal	Unknown (LGS)	L CMN, R HCP
F11	11	Y (R TL and disconnection)	N	Focal	Unknown	R ANT, L HCP
M28	27	Y (Partial R T resection)	N	Focal	Structural (Polymicrogyria)	R CMN, R P
F25	24	Y (L FP resection)	N	Focal	Structural (Tuberous Sclerosis)	L CMN, L F
M31	17	N	N	Focal	Structural (Post-traumatic)	L CMN, L P operculum
F47	28	Y (L F resection)	N	Focal	Unknown	L CMN, L post F
F44	39	Y (R ATL)	N	Focal	Unknown	L CMN, L T
M28	25	N	Y	Focal	Structural (pathogenic variant SPAST)	L ANT, L T
M23	23	Y (partial F and T resections)	Y	Focal	Unknown	R ANT, R posterior central region
M24	9	N	N	Focal	Unknown	L ANT, L P

ATL, anterior temporal lobectomy; ANT, anterior nucleus of the thalamus; B, bilateral; CC, corpus callosotomy; CMN, central median nucleus; F, frontal; FP, frontoparietal; Hippo, hippocampus; L, left; LGS, lenox-gastaut syndrome; MTS, mesial temporal sclerosis; P, parietal; R, right; and T, temporal.

* R frontal lobe resection, CC, VNS, anterior comissurotomy L temporal lobectomy L parietal-occipital disconnection L orbital-frontal resection.

### 3.2. Seizure characteristics

Baseline seizure frequency ranged from 3 to 2,250 disabling seizures per month (median 15). All patients had seizures captured on scalp EEG. Five patients had seizures with a generalized onset and 20 patients had seizures with focal onset. Of the patients with focal onset, 11 had greater than three foci, four had two foci, and five had a single focus. Onset zones on scalp were varied and as follows: Frontal (12), parietal (7), occipital (4), temporal (13), and mesial temporal (2). The majority had an intracranial EEG (20). Onsets on intracranial EEG were slightly different from prior scalp EEG and as follows: Frontal (12), parietal (10), occipital (4), temporal (9), mesial temporal (7), and insula (2). Two of the generalized onset patients had juvenile myoclonic epilepsy, two had Lennox Gastaut Syndrome and one likely had generalized epilepsy with tonic clonic seizures alone. Three of the patients with generalized onset underwent an intracranial EEG prior to RNS placement (Table 1).

### 3.3. Location of implant, detection and stimulation

Twelve patients had at least one lead placed in the CMN, 13 had at least one lead placed in the ANT. Four patients had bilateral thalamic depths and 21 patients had unilateral thalamic depths with another RNS strip or depth in various regions throughout the brain (Table 1). The thalamic lead(s) were used for detection in 18 patients whereas detection was solely non-thalamic in seven patients. Stimulation was delivered on the thalamic depths in 24 patients. In one patient, the thalamic lead was used for detection only and not stimulation.

### 3.4. Seizure reduction

**Figure 2:**
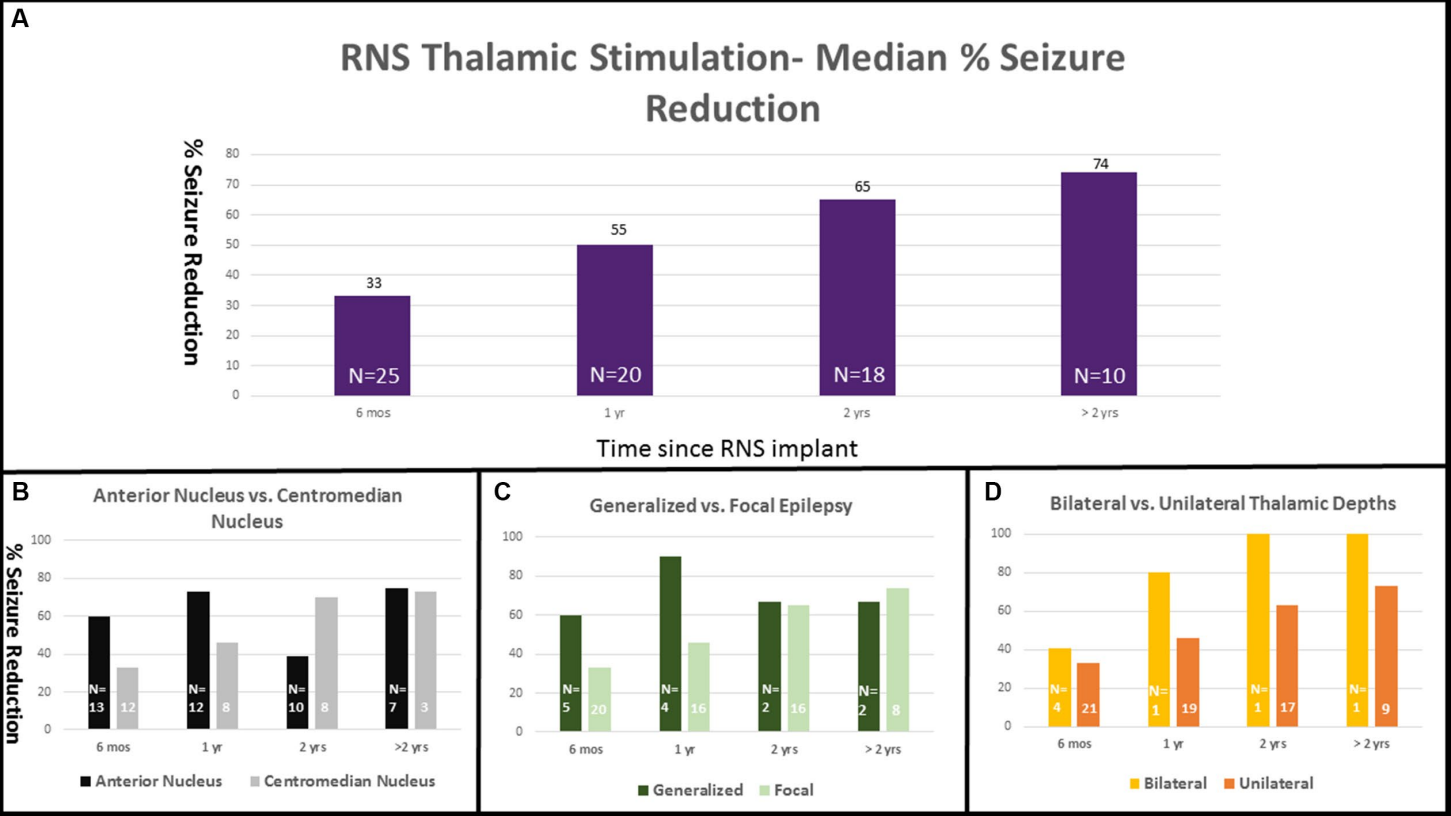
RNS thalamic stimulation—Median % Seizure Reduction with Subgroup Comparison. Percent seizure reductions was 33% at 6 months (*n* = 25), 55% at 1 year (*n* = 20), 65% at 2 years (*n* = 18), and 74% at >2 years (*n* = 10; **A**). Percent response is separated into subgroup comparisons with anterior nucleus vs. centromedian nucleus **(B)**, generalized vs. focal epilepsy **(C)**, and unilateral vs. bilateral thalamic depths **(D)**.

For patients with thalamic depths there was a median seizure reduction of 33% at 6 months, 55% at 1 year, 65% at 2 years, and 74% at >2 years (Figure 2A). Twenty-one patients (84%) reported a reduction of seizures at every visit. Nine patients reported at least 3 months of seizure freedom. Overall, eight patients (32%) reported an interval of at least 6 months with a greater than 90% seizure reduction. One patient reported worsened seizure frequency at 2 years and at the most recent visit. Three patients reported no change in seizure frequency at every follow up visit.

**Figure 3:**
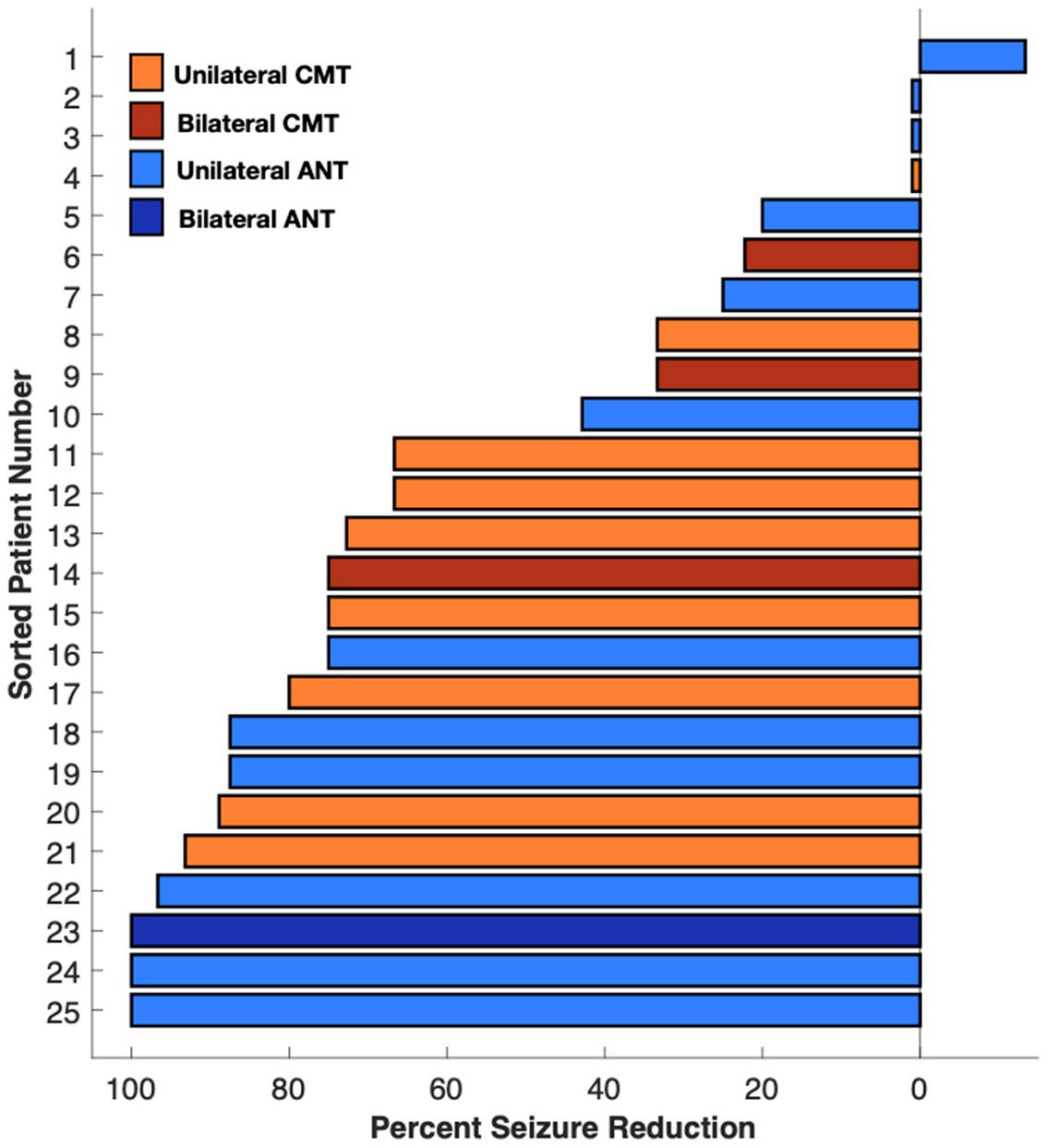
Median percent seizure reduction in ANT, CMN, bilateral, and unilateral depths at most recent visit.

Seizure reduction was further analyzed based on lead location (ANT vs. CMN), type of epilepsy (generalized vs. focal), and unilateral vs. bilateral thalamic stimulation. Those with ANT leads (13) had median reductions of 60, 74, 39, and 75% at 6 months, 1 year, 2 years, and > 2 years respectively, compared with a median reduction of 33, 46, 70, and 73% in the CMN group (12) (Figure 2B). The single patient who experienced a worsened seizure frequency had an ANT lead. The seizure reduction in the ANT group was more heterogeneous including the patient with worsened seizure control at the 2 year mark, two patients who did not show any change and two patients who were super responders with 100% seizure control at the most recent follow up. Those with generalized onset epilepsy (5) had a median seizure reduction of 60, 90, 67, and 67% at 6 months, 1 year, 2 years, and > 2 years compared to reductions of 33, 46, 65, and 74% in the focal epilepsy group (20) (Figure 2C). Patients with bilateral thalamic leads (4) experienced a median seizure reduction of 41, 80, 100, and 100% at 6 months, 1 year, 2 years, and > 2 years compared to median seizure reduction of 33, 50, 63, and 73% in the unilateral (21) group (Figure 2D). Seizure reduction at the most recent visit is summarized in Figure 3.

### 3.5. Anti-seizure medications

**Figure 4:**
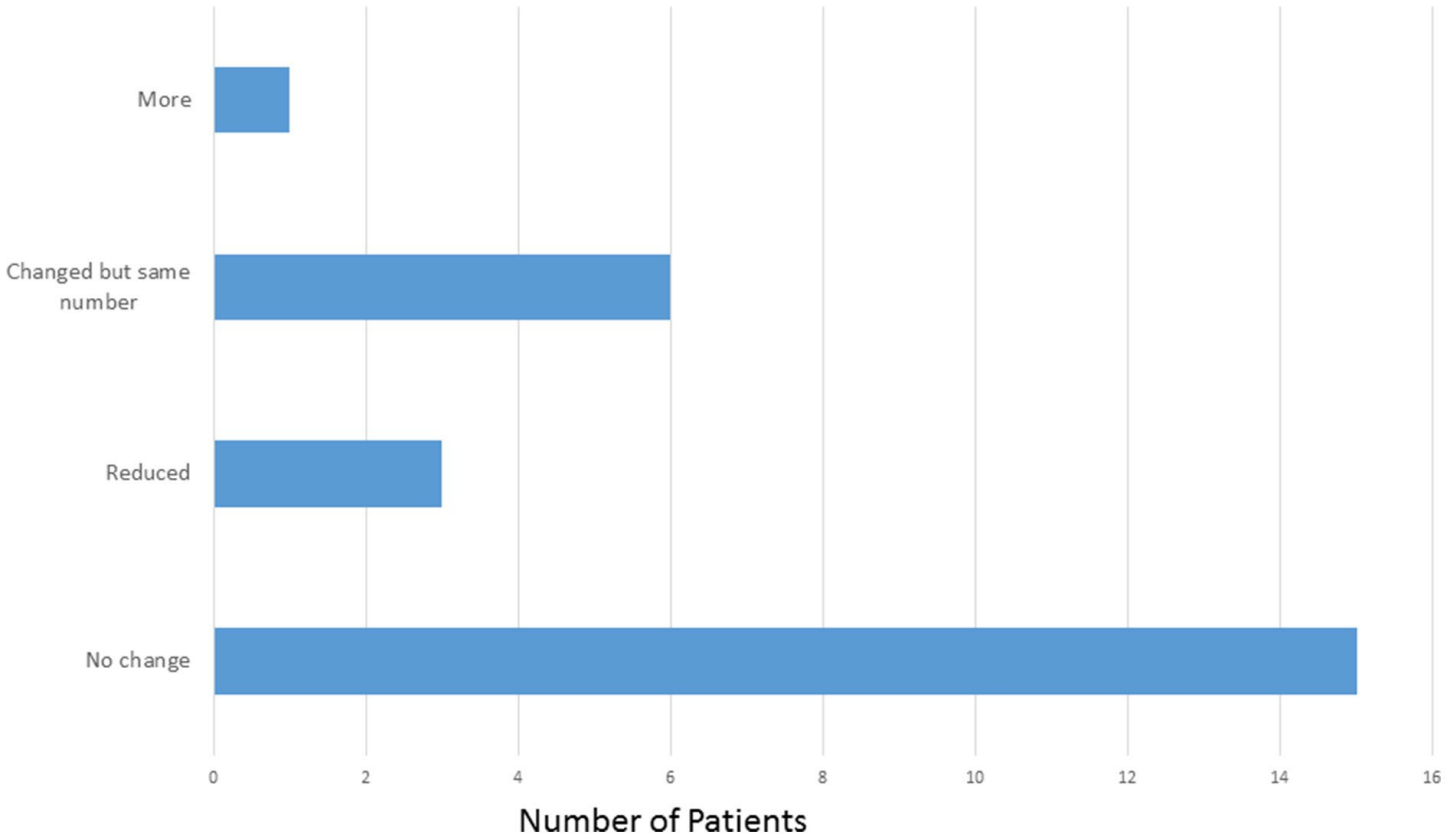
Anti-seizure medication changes with thalamic stimulation.

The mean number of ASMs at the start of the study, prior to implant was 2.64 compared with 2.52 at the most recent visit. Of the 25 patients, 15 (60%) patients did not have a change in the number or type of ASMs. Three patients (12%) were on reduced ASMs at the most recent visit. Six patients (24%) had medication adjustments which resulted in the same number of ASMs and one patient (4%) was on one more ASM at the most recent visit compared to prior to implant (Figure 4).

### 3.6. Stimulation side effects and serious adverse events

No stimulation side effects outside of the clinic were reported. Two patients had events that qualified as serious adverse events: One patient had asystole within the first 6 months of having the RNS implanted, likely caused by a seizure, followed by syncope resulting in a hospitalization. This was unlikely to be related to the RNS device. A second patient had a left intraventricular hemorrhage at the time of implant noted on an intraoperative MRI and post-operative CT. This was asymptomatic without chronic sequelae. The patient left the hospital on post-operative day 2 in good condition.

### 3.7. Global clinical impression

The GCI-I was assessed for all patients by the care team. This assessment evaluates the overall quality of life. Nine patients were very much improved, six patients were much improved, seven patients were minimally improved, and three patients had no change. No patients assessed were clinically worse post-RNS.

## 4. Discussion

The thalamic nuclei with their widespread connections across cortical and subcortical regions hold promise to exert multi-focal or global influence on the brain, if harnessed properly. Original reports of thalamic stimulation and its effect on seizures date back to cat and human models in the 1950 and 1960s (28–30). Chronic stimulation in the thalamus has been shown to cause as well as abort seizure activity. Specific thalamic nuclei and their wider connections to other brain regions have been widely investigated. The CMN is an “intralaminar” nucleus that broadly affects the cortex (sensorimotor, premotor) with prominent connections to the cerebellum and basal ganglia and is likely involved in arousal and attention (31–33). Meanwhile, the ANT is part of Papez circuit which links medial frontal cortex with medial temporal lobe structures and is believed to underlie aspects of emotional and mnemonic function (34, 35). Up until now, several case reports of ANT and CMN responsive neurostimulation have been published in the literature (1–14). Reports on neuromodulation involving other thalamic nuclei, including the pulvinar, have been examined as well (36, 37). To date this case series is the largest involving the CMN and ANT. While there are no randomized controlled data comparing nucleus selection for thalamic stimulation in epilepsy, the current convention is to select the thalamic nucleus with connections most involved in the patient’s epilepsy network. For example, ANT was often selected based on limbic involvement and the CMN where motor/frontal involvement seemed most prominent. The working theory is that the thalamic stimulation, given at the onset of a potential seizure, can de-synchronize the electrical activity by spreading to areas involved in the seizure network.

The patients included in this study should be conceptualized as among the most refractory patients. The choice of nuclei selected was based on seizure semiology and electrographic seizure signature. When an extensive seizure network is involved, thalamic neuromodulation appears the most attractive option after medications and in many instances, previous resections have failed.

In this multicenter review, the 25 RNS patients with thalamic RNS leads had seizure reduction profiles similar to that of the larger group of patients with cortical (non-thalamic) RNS studied in the long-term prospective open label trial (38). Here, at the time of the >2 year visit the median seizure reduction was 74%. In the long-term prospective trial, the median reduction at 9 years was 75%. Similarly, DBS in the ANT was shown to have a 75% median seizure reduction at 10 years (27). RNS in the thalamus appears to be a safe and well tolerated procedure. One of the two patients with serious adverse events was unrelated to the device and the other was an asymptomatic intraventricular hemorrhage. Thalamic RNS seems to have few side effects and no long term cognitive or mood changes were observed. In fact, most patients showed a general improvement in overall functioning.

## 5. Limitations

This study has several important limitations: this was a retrospective review with a relatively small number of heterogeneous patients and there was no blinded period or placebo control (AKA sham stimulation period). Parameters of stimulation pathway, stimulation duration, and stimulation intensity were not pre-set and were up to the individual clinician or group. Furthermore, seizure frequency was determined by patient report and expert clinician assessment, rather than by device-based measurements, (serial RNS-based measures of seizure frequency are typically confounded by changes in detection thresholds during programming visits). These factors prevent a more objective and controlled analysis of seizure reduction in this retrospective series. The seizure etiology and target nucleus of the thalamus varied among this heterogeneous cohort, limiting power for subgroup analysis in this initial case series. We included specific outcome data for the subgroups (bilateral vs. unilateral stimulation, ANT vs. CMT, generalized vs. focal epilepsy). While these numbers are far too small to be significant, we believe these are important subgroups to consider in future prospective study design.

Additionally, most patients (21/25) had thalamic and non-thalamic stimulation. It is not known what portion of the benefit came from the thalamic stimulation. The purely thalamic stimulation group was only four patients (the bilateral thalamic subgroup) and therefore not large enough for substantive conclusions. While the GCI-I tool was used to obtain a gross assessment of overall functioning, more detailed and validated neuropsychological tools would be of significant benefit to measure mood and cognitive functioning with greater precision.

Overall, this work suggests that RNS treatment in the thalamus is safe and effective at reducing seizure frequency and improving quality of life in patients with difficult seizure types that often would not typically be amenable to further neurosurgical intervention. However, larger, prospective studies with stricter controls and assessments are needed to determine optimal treatment strategies for this highly refractory group of patients.

## Author’s note

CS conducted the research as an epilepsy fellow at Icahn School of Medicine at Mount Sinai.

## Data availability statement

The raw data supporting the conclusions of this article will be made available by the authors, without undue reservation.

## Ethics statement

The studies involving human participants were reviewed and approved by IRB at Mount Sinai Hospital, NYU Langone Medical Center, Emory University, Yale University, Brown University/Rhode Island Hospital, Corewell Health, and Medical College of Wisconsin. Written informed consent from the participants’ legal guardian/next of kin was not required to participate in this study in accordance with the national legislation and the institutional requirements.

## Author contributions

MF, LM, CS, and PD conceptualized the project. OE, MF, and CS constructed the database and questionnaire. MF, LM, and CS drafted the manuscript. All authors contributed to the article and approved the submitted version.

## Conflict of interest

KB receives salary support from Neuropace. FP and LM are a speaker for Neuropace.

The remaining authors declare that the research was conducted in the absence of any commercial or financial relationships that could be construed as a potential conflict of interest.

## Publisher’s note

All claims expressed in this article are solely those of the authors and do not necessarily represent those of their affiliated organizations, or those of the publisher, the editors and the reviewers. Any product that may be evaluated in this article, or claim that may be made by its manufacturer, is not guaranteed or endorsed by the publisher.
